# A perspective: A biochemical model of photosynthetic CO_2_ assimilation in leaves of C_3_ species (Planta 149, 78–90)

**DOI:** 10.1007/s00425-025-04834-7

**Published:** 2025-10-06

**Authors:** Susanne von Caemmerer, Joseph A. Berry, Graham D. Farquhar

**Affiliations:** 1 Division of Plant Sciences, Research School of Biology, The Australian National University, Acton, ACT 2600 Australia; 2 Department of Global Ecology, Carnegie Institution of Washington, Stanford, CA 94305-4101 USA

## Abstract

The model of C_3_ photosynthesis of Farquhar et al. (1980) integrated knowledge of the functioning of the biochemical components of photosynthetic carbon assimilation in C_3_ plants. The model linked equations describing activated Rubisco kinetics with those on the stoichiometry of the photosynthetic carbon reduction cycle and the photorespiratory carbon oxidation cycle, particularly on their energetic (electron transport, ATP synthesis and NADPH) requirements. It included temperature dependencies of these processes and combined them with a semi-empirical equation for the dependence of potential electron transport rate on absorbed irradiance. The model aimed to match generalized observations of photosynthetic gas exchange of leaves with predictions from this mathematical summary of photosynthesis. In this model, we introduced the hypothesis that the rate of Rubisco carboxylation could not exceed the capacity for RuP_2_ regeneration or the enzymatic capacity to consume RuP_2_, and that the system behaved as a teeter-totter (see-saw) with a sharp transition from one limiting state to the other. We suggested that to model genotypic and phenotypic variations amongst leaves most parameters could be assigned a priori and only the maximum Rubisco activity (*V*_*cmax*_) and the maximal electron transport rate, *J*_*max*_, needed to be varied. This simplicity of use led to the wider spread application and success of the model.

## Introduction

The 1970’s was a pivotal era for examining photosynthesis with the relatively new tool of gas exchange measurements, which linked CO_2_ assimilation rates to changes in CO_2_, irradiance and temperature. A great body of work was accumulating looking at CO_2_ uptake and water loss of leaves under different environmental conditions (e.g. Björkman et al. 1972). At the same time, biochemical studies of Rubisco led to vital new understandings. For example, George Bowes and Bill Ogren discovered that O_2_ was a competitive inhibitor of CO_2_ fixation by Rubisco and also an alternative substrate leading to a side reaction that fuelled photorespiration (Bowes and Ogren 1972). The insight that Rubisco required CO_2_ and Mg^+^ bound in its active site before it was catalytically competent facilitated the first reproducible measurements of its kinetic constants ( Laing et al. 1974; Lorimer et al. 1976; Badger and Collatz 1977). The release and refixation of ammonia during photorespiration was important for capturing the energetics of photorespiration (Keys et al. 1978; Woo et al. 1978).

**Fig. 1:**
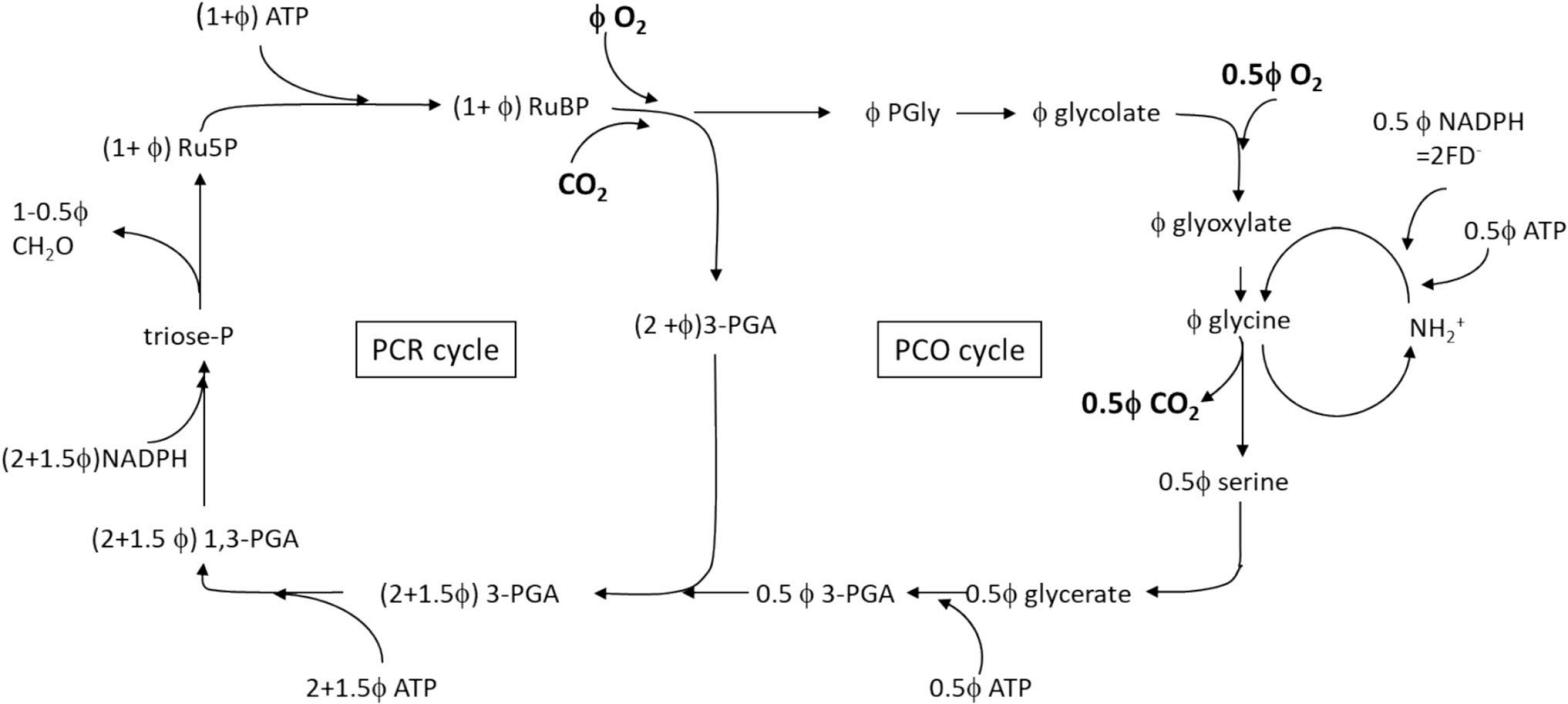
Simplified photosynthetic carbon reduction (PCR) and photorespiratory carbon oxidation (PCO) cycles. The symbol *ϕ* denotes the ratio of the rate of Rubisco oxygenation to carboxylation rate, *V*_o_/*V*_c_. Note that the regeneration of 0.5 mol of PGA from 1 mol PGly requires 0.5 mol ATP. It also includes the release and refixation of 0.5 mol of ammonia, which requires 1 mol of reduced ferredoxin. In terms of electron transport, this is equivalent to 0.5 mol of NADPH and 0.5 mol of ATP (Keys et al. 1978; Woo et al. 1978). The stoichiometry and diagram are redrawn from von Caemmerer (2020) based on Farquhar et al. (1980)

The C_3_ photosynthetic model developed by Farquhar von Caemmerer and Berry captured this biochemical understanding and linked equations describing Rubisco kinetics with the stoichiometry of the carbon reduction cycle (PCR) and photorespiratory carbon oxidation cycle (PCO) (Fig. 1)**.** It included temperature dependencies and a semi-empirical equation for the dependence of potential electron transport rate on absorbed irradiance and built on the pioneering modelling of Hall (Hall and Björkman 1975; Hall 1979), Tenhunen (Tenhunen et al. 1979), Peisker (Peisker 1974, 1976), Laisk (Laisk 1970, 1977) and others. Our model was built around the observation that Rubisco controls the flow of metabolites to the PCR and PCO cycles (Fig. 1). These cycles regenerate ribulose bisphospate (RuP_2_) to sustain CO_2_ fixation following carboxylation or oxygenation events. We realized that we could write two equations for the rate of Rubisco carboxylation, *V*_*c*_, based on the newly available kinetics of Rubisco. In one case, *V*_*c*_ would be controlled by the availability of energy (NADPH and ATP) (*V*_*c*_ = *J’*), and in the second case if the supply of energy becomes non-limiting, then *V*_*c*_ would be determined by the RuP_2_-saturated rate of Rubisco to catalyze carboxylation and oxygenation (*V*_*c*_ = *W*_*c*_). Further, we envisioned that control of *V*_*c*_ could switch from one state to the other depending on whether light was limiting or saturating for photosynthesis. Thus, we could write ${V}_{c}=min\left\{{W}_{c},{ J}^{\prime}\right\}$*.* This provided the framework of our model, with the addition of an expression for the rate of electron transport as a function of irradiance. [We found this “teeter-totter” or “see-saw” approach worked in an earlier version of the model (Berry and Farquhar 1978)]. However, we recognized that this assumption was problematic given classical Michaelis–Menten kinetics for a two-substrate reaction. It was possible that occupancy of the RuP_2_ binding site might influence the binding constants of the other substrates (CO_2_, and O_2_), and the hyperbolic approach to saturation typically seen in enzyme assays would require a large change in the pool of RuP_2_ for Rubisco activity to saturate in vivo.

**Fig. 2:**
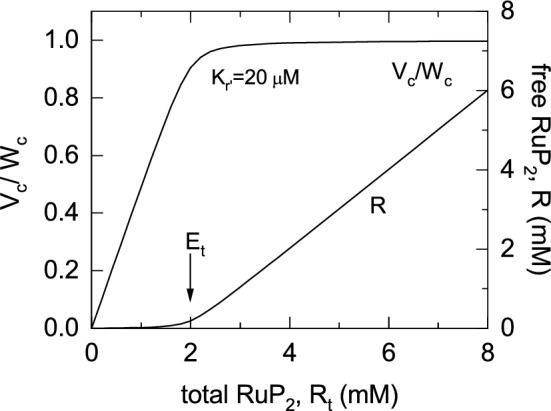
Modelled Rubisco carboxylation rate *V*_*c*_ (expressed as a fraction of the ribulose bisphospate, RuP_2_, saturated carboxylation rate *W*_*c*_) as a function of free and enzyme-bound RuP_2_, *R*_*t*_. The curve is approximated by the equation ${V}_{c}/{W}_{c}=min \left\{\left[{R}_{t}\right]/\left[{E}_{t}\right],1\right\}$. The enzyme site concentration, *E*_*t*_, is 2 mM and the apparent *K*_m_ for RuP_2_, *K*_*r’*_ = 20 µM. Also shown is free RuP_2_, *R*. The diagram is modified from von Caemmerer (2000). Experimental verification of this concept was provided by Mott et al. (1984)

**Fig. 3:**
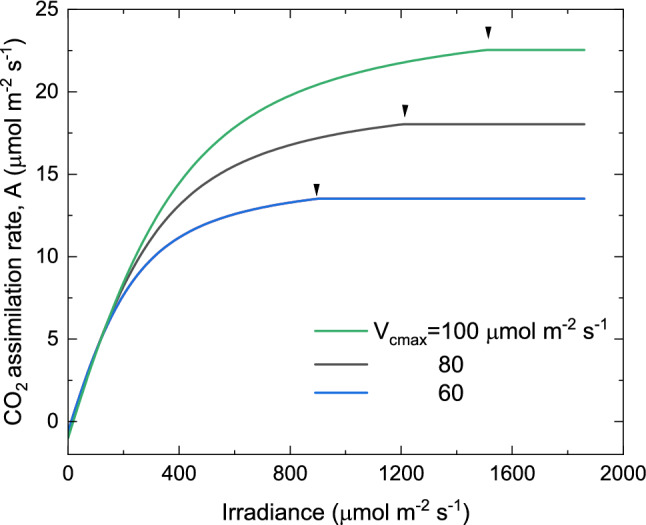
The modelled rate of CO_2_ assimilation versus irradiance at three levels of Rubisco carboxylase activity. The maximal electron transport rate was 1.725 times maximal Rubisco activity *V*_*cmax*_. Day respiration was 0.01 *V*_*cmax*_ and chloroplast CO_2_ partial pressure = 220 µbar at a leaf temperature of 25 °C. Parametrization of the model used updated kinetic constants of Rubisco given by von Caemmerer (2000). Arrows indicate transition from electron transport to Rubisco limitation

Graham conducted a critical review of the kinetics of Rubisco to address these issues (Farquhar 1979). He concluded that the reaction mechanism was probably ordered with RuP_2_ binding first and that bound RuP_2_ was most likely the binding site for CO_2_ or O_2_. Thus, interaction between RuP_2_ concentration and the Michaelis–Menten constants for the gaseous substrates was unlikely. His analysis also explored the RuP_2_ control of the rate of reaction, given that the concentration of Rubisco active sites (E_t_) in vivo far exceeds the apparent Michaelis constant for RuP_2_. Consequently, most of the RuP_2_ present in the stroma should be bound to Rubisco active sites when RuP_2_ regeneration is limiting, and the rate should increase with total (bound + free) RuP_2_ concentration until abruptly saturating when the total RuP_2_ concentration reaches the concentration of active sites yielding the response curve shown in Fig. 2. Leaf specific correlations between in vitro measurement of *V*_*cmax*_ and *J*_*max*_ provided a quantitative basis for understanding differences in photosynthetic properties of plants adapted to different environments, or receiving different nutrition (Fig. 3, von Caemmerer and Farquhar 1981).

## Key findings of the paper

1. Succinct description of the PCR and PCO cycles with stochiometric links to ATP and NADPH consumption. These are described in Fig. 1. The guiding principle of the model design was to think carefully about what could safely be left out.
2. Recognising Rubisco’s special in vivo kinetics (Farquhar 1979). This was based on the realisation that Rubisco was present in high concentrations in the chloroplast and that, therefore, in vivo Rubisco kinetics with respect to free and enzyme-bound RuP_2_ did not follow the standard Michaelis–Menten kinetics but could be described by equations for a tight-binding inhibitor or substrate (Fig. 2).
3. Succinct formulation of the key equation: ${V}_{c}=min \left\{{W}_{c},{ J}{^\prime}\right\}$, which described Rubisco carboxylation as either limited by its RuP_2_ saturated rate, *W*_c_, or *J’*, the maximum carboxylation rate allowed by the electron transport. This was inspired by Rubisco’s special in vivo kinetics shown in Fig. 2.
4. The model could reproduce common gas exchange features such as the dependence of quantum yield on [CO_2_] and temperature, which had been quite puzzling. The dependence of the CO_2_ compensation point (the [CO_2_] where there is no net CO_2_ flux in or out of the leaf) as a function of temperature, and the temperature dependence of CO_2_ assimilation rate, *A*, at different irradiances and [CO_2_].
5. It could also model the effect of differences in leaf nitrogen content, by varying the *V*_cmax_ and *J*_*max*_, mimicking the results of *Atriplex patula* grown at different irradiances (Fig. 3, Björkman 1972)*.*
6. This established that to model genotypic and phenotypic variations amongst leaves, most parameters could be assigned a priori and only maximum Rubisco activity, *V*_*cmax*_*,* and maximum electron transport rate, *J*_*max,*_ needed to be adjusted. This was experimentally tested by von Caemmerer and Farquhar (1981)

## Contribution to current research

### Rubisco is at the heart of photosynthesis modelling

Fundamentally, the model is still used as originally designed, but some modifications have been suggested (see discussion below). The kinetic parameters of Rubisco have been updated using in vivo and in vitro measurements of tobacco Rubisco (von Caemmerer et al. 1994; Bernacchi et al. 2002), however, these may not suit all species and more work is needed on this aspect (Sargent et al. 2024; Silva-Pérez et al. 2017) One of the puzzles at the time was that we needed to assume that Rubisco was fully active at high light to be able to quantitatively account for CO_2_ assimilation rates. To function, Rubiso’s active sites must be activated by the reversible carbamylation of a lysine residue within the active site, followed by rapid binding of an essential Mg^2+^ (Andrews and Lorimer 1987). In vitro studies of the activation process of Rubisco suggested that Rubisco would be at most 25% active at pH, Mg^2+^ and [CO_2_] thought to occur in the chloroplast (Lorimer et al. 1976). von Caemmerer and Edmondson (1986) followed this up and gas exchange measurements combined with rapid freeze clamping of leaves confirmed that Rubisco was indeed fully activated at high light. Experiments by Mott et al. (1984) and von Caemmerer and Edmondson (1986) also confirmed that RuP_2_ was saturating at low [CO_2_] and low at high [CO_2_]. The puzzle of how Rubisco is fully activated in vivo was solved with the discovery of Rubisco activase protein in an Arabidopsis mutant that had poorly activated Rubisco in the light (Salvucci et al. 1985; Portis 1990; Salvucci and Ogren 1996). Studies then showed that activase, requiring ATP hydrolysis, acts to remove sugar phosphates from carbamylated and uncarbamylated Rubisco sites thus promoting carbamylation (Portis 1990; Andrews et al. 1995). The interaction between Rubisco and Rubisco activase remains an active research field (Portis et al. 2008) and the regulation of Rubisco activase has been targeted for crop improvement (Parry et al. 2013; Carmo-Silva et al. 2015; Scafaro et al. 2019).

### CO_2_ assimilation rate and electron transport capacity

Farquhar et al. (1980) proposed two equations for the electron transport-limited CO_2_ assimilation rate, one based on the NADPH and the other based on the ATP requirement. The first equation has remained unchanged, but it is now generally accepted that the production of ATP requires four rather than three protons and that the Q-cycle is operational ( von Caemmerer 2000; Yin et al. 2004; Johnson and Berry 2021). Yin et al. (2004) have extended the equations for the electron transport limited rate, including terms of cyclic and pseudo cyclic electron transport rate. Recently, Johnson and Berry (2021) proposed a new model that replaces *J*_*max*_ and the empirical equation for electron transport in the Farquhar et al. (1980) model with a more mechanistic model of electron transport. It does not fundamentally change the form of the equation for the rate of CO_2_ assimilation but aids mechanistic understanding of *J*_*max*_. It provides details on how the CO_2_ assimilation rate is related to the dynamics of electron transport through photosystem I and photosystem II and provides a link between carbon metabolism and chlorophyll fluorescence. One of the greatest technical advances has been the use of pulse-modulated chlorophyll fluorescence measurements to estimate the rate of electron transport (Schreiber et al. 1986; Genty et al. 1989). Many portable gas exchange systems now routinely measure leaf chlorophyll fluorescence at the same time as gas exchange. Linear chloroplast electron transport rate can be directly calculated from the quantum yield of photosystem II obtained from chlorophyll fluorescence (Genty et al. 1989). Chlorophyll fluorescence provides an excellent way to distinguish RuP_2_ regeneration limitation from the Rubisco limited CO_2_ assimilation rate as chloroplast electron transport calculated from fluorescence becomes independent of [CO_2_] when RuP_2_ regeneration limits CO_2_ assimilation rate (von Caemmerer et al. 2009). However, it must be recognised that gas exchange measures the whole leaf in the chamber whereas chlorophyll fluorescence only measures a certain cell layer.

### Further model developments

Harley and Sharkey (1991) introduced a third limitation to CO_2_ assimilation rates at high intercellular CO_2_, *C*_*i*_ called a triose phosphate, TP, limitation. It has been added to explain the sometime less-than-expected increase in *A* from a *J* limitation at high *C*_i_ (Harley and Sharkey 1991; Sharkey et al. 2007). This limitation is due to the restriction set by the rate at which triose phosphates are utilized in the synthesis of sucrose or starch. As the use of triose phosphates is stoichiometrically exchanged with the release of phosphate during sucrose or starch synthesis. A limitation in TP utilization (TPU) can result in a limitation to photophosphorylation, and, thus, to RuBP regeneration. It is important to acknowledge this limitation as otherwise fitting of *A*/*C*_i_ curves, the response of CO_2_ assimilation rate, *A*, to intercellular CO_2_ partial pressure, *C*_*i*_, can underestimate electron transport capacity *J*_*max..*_TPU limitations are variable and not always present (for review see, Yin et al. 2021). A further modification has been the introduction of a photorespiration-associated nitrogen and C1 metabolism. This modification proposes that a fraction of glycolate carbon is taken out from the photorespiratory pathway as glycine and serine (Busch et al. 2018; Busch 2020). The result of this modification is a decrease in the compensation point, but further experimental verification is required. At the time Farquhar et al. (1980) built the C_3_ photosynthesis model they assumed that the chloroplast [CO_2_] was similar to the intercellular [CO_2_]. It has now been realised that is not the case particularly at high rates of CO_2_ assimilation. Mesophyll conductance, *g*_*m*_*,* which quantifies the ease with which CO_2_ diffuses from intercellular airspace within a leaf to the sites of Rubisco carboxylation within chloroplasts is now frequently measured using combined measurements of gas exchange and carbon isotope discrimination (Evans et al. 1986). Detailed equations incorporating g_m_ and the above modifications have all been reviewed by Yin et al. (2021).

### From leaf to canopy and the globe

To model photosynthesis at the canopy and global scales it is important to link CO_2_ assimilation rate of leaves with the inevitable rate of water loss (Farquhar and Sharkey 1982). Wong et al. (1979) showed that under many conditions, the ratio of intercellular and ambient CO_2_ (*C*_*i*_*/C*_*a*_) is conserved but reduces with increasing leaf to air humidity difference. This led to the development of a succinct relationship between stomatal conductance and the rate of CO_2_ assimilation (Ball et al. 1987). This has been combined with the photosynthesis model to provide information to canopy photosynthesis models (Wu et al. 2019). Other approaches have used optimisation theories of carbon gain per water loss (Cowan 1986). These ideas have been incorporated in terrestrial models of carbon uptake (Medlyn et al. 2011, 2013). However, there is still insufficient information for a mechanistic model of stomatal function. Buckley (2017) provides an update on stomatal modelling.

Farquhar et al. (1980) scaled up from the chloroplast to the leaf assuming uniformity of light, but acknowledged the light distribution within the leaf provided a cause of uncertainty. Terashima and colleagues explored the gradients of light and photosynthetic capacity within leaves (Terashima and Inoue 1985; Terashima and Saeki 1985). These finer details are rarely incorporated into modelling approaches. To model canopy photosynthesis, several approaches have been taken. Models that treat the canopy as a big leaf tend to overestimate canopy photosynthesis (de Pury and Farquhar 1997). Other models have incorporated the Farquhar et al. (1980) model into canopy models that divide the canopy into layers (Humphries and Long 1995; Song et al. 2017). The third approach has been to divide canopy leaves into shade and sun leaves. This approach succinctly captures canopy photosynthesis in a simpler mathematical format which has now been integrated into crop growth models (de Pury and Farquhar 1997; Wu et al. 2019). Earth system models of terrestrial photosynthesis have also incorporated the Farquhar et al. (1980) model. One of the biggest challenges is accurate temperature dependencies at very low and very high temperature. Furthermore, plants are known to acclimate to different growth temperatures and all this needs to be understood and incorporated into terrestrial biosphere models to provide robust projections of global change (Berry and Björkman 1980; Rogers et al. 2017).

## Data availability:

Not applicable.
